# The therapeutic potential of microdosing psychedelics in depression

**DOI:** 10.1177/2045125320950567

**Published:** 2020-08-27

**Authors:** Kim P.C. Kuypers

**Affiliations:** Department of Neuropsychology & Psychopharmacology, Faculty of Psychology & Neuroscience, Maastricht University, PB 616, 6200 MD, the Netherlands

**Keywords:** depression, LSD, microdosing psychedelics, psilocybin

## Abstract

Microdosing psychedelics is the repeated use of small doses of, for example, lysergic acid diethylamide (LSD) and psilocybin, typically for a few weeks. Despite the popular and scientific attention in recent years, and claims by users that it has therapeutic value in affective disorders like depression, little scientific knowledge is available to back this. The purpose of this review was to investigate whether there are scientific grounds to state that this practice could be helpful in the treatment of affective disorders, and safe to use repeatedly. To that end, the literature (PubMed, MedLine) was searched, looking for (controlled) experimental studies with low doses of LSD and/or psilocybin, in healthy volunteers and patient samples. After a selection process and the addition of relevant articles, 14 experimental studies entered this review. Findings show that both LSD (10–20 mcg) and psilocybin (<1–3 mg) have subtle (positive) effects on cognitive processes (time perception, convergent and divergent thinking) and brain regions involved in affective processes. Besides the pleasant experience, increased anxiety and a cycling pattern of depressive and euphoric mood were also found. With regard to safety, it was demonstrated that low doses are well tolerated (in healthy volunteers) and have no-to-minimal effects on physiological measures. While it is yet unclear whether psychedelic microdosing is of therapeutic value for depression, the aforementioned effects on selective processes suggest that low doses of psychedelics could play a role in depression by inducing some kind of cognitive flexibility, which might lead to decreased rumination. While previous studies were conducted mostly in small samples of healthy volunteers, future placebo-controlled clinical trials in depressed patients are required to understand the therapeutic value of microdosing psychedelics, how this differs from therapy using full psychedelic doses, and whether different psychedelics have different effect patterns. The proposed research will give new insights into the potential of future alternative psychiatric treatment forms that are fiercely needed.

## Introduction

Lysergic acid diethylamide (LSD) and psilocybin are prototypical classical psychedelics. These psychoactive substances typically produce perceptual distortions and mind-altering effects, mainly by agonistic action at the serotonin (5-HT) 2A brain receptor.^1^ Recent placebo-controlled experimental studies have also shown that LSD and psilocybin increase self-rated positive mood and social behaviour, enhance emotional empathy, and reduce recognition of negative emotional states (e.g. sadness and fear).^2–4^ Oral doses typically used in human research, and producing the aforementioned effects, are 100–200 mcg LSD, usually given as fixed dose, and 15 mg of psilocybin, on average, which is usually dosed per body weight.^2,4^

Psychedelics are seen as a class of substances scoring relatively high on physiological and psychological safety when used under supervision in a controlled setting.^1,5^ In general, they do not induce dependence, or adverse effects that would not be manageable when given in appropriate doses, and in the presence of someone who can provide psychological support, if needed.^1,4,6^ Moreover, preliminary findings and anecdotal reports suggest that psychedelics even show therapeutic potential in substance use disorders.^1,7,8^ In addition, current research is investigating therapeutic applications of these substances in psychiatric disorders, with a focus on affective disorders.^8–11^ While the intensity or quality of the psychedelic experience seems to contribute to its therapeutic effect,^12^ anecdotal evidence also suggests that repeated use of smaller doses without the psychedelic experience (‘microdosing’) is efficacious self-treatment for people suffering from affective disorders.^13,14^

In general, a microdose is considered to be one tenth of a dose normally causing hallucinogenic effects. When taking the doses used in clinical research as a reference,^2,4^ a microdose then would be 10–20 mcg of LSD and/or 0.3–0.5 g of psilocybin-containing mushrooms.^15,16^ In a recent survey, users reported taking between 6 and 20 mcg LSD and 0.2–0.5 g of dried psilocybin mushrooms^13,17,18^ with a microdosing frequency that ranges between 2 and 4 times a week, this for a few weeks, to months, or even years, although the latter is rare.^15,18^ Reported short-term benefits of microdosing include an increase in positive mood, a decrease in negative mood, and in improvement relationships with others and their environment,^15,17–21^ which seems to be in line with the effects of full psychedelic doses, though without the perceptual effects. Interestingly enough, users sometimes attribute different effects to the different substances where LSD is more associated with cognitive and/or stimulating effects and psilocybin with emotional or well-being effects.^17,22^ This stronger stimulating character of LSD compared with psilocybin was seen by some as a plus, while others experienced it as uncomfortable.^17,22^ Of note, future research needs to elucidate whether the higher affinity of LSD, compared with psilocybin, for the dopaminergic receptors explains this stimulant effect, and/or whether this is (partly) due to expectancy bias.^23^

Next to positive effects, acute negative effects do occur when individuals are under the influence of these psychedelics including psychological (‘increased anxiety’) or physiological (‘discomfort’) changes.^15,18^ Interestingly, this increased anxiety is suggested to be linked to the surface emergence of latent emotional content by microdosing. Along the same lines, it is reasoned that this could accelerate a healing process in a therapeutic context because these emotions can then be used.^22^ Interestingly, Albert Hofmann, the ‘discoverer’ of LSD and its hallucinogenic effects, stated decades ago that ‘very small doses, perhaps 25 micrograms’, could be useful as an antidepressant.^24,25^ This seems to be confirmed in the reports of people self-treating with microdoses of psychedelics to combat symptoms of affective disorders such as depression and anxiety disorders.^14,26^

Despite the positive claims of microdosers who self-treat their conditions, no clinical trial to date has focused on the question whether (repeated) administration of psychedelics in low doses can serve therapeutic potential in affective disorders. The aim of the present review was to investigate, based on findings from (controlled) experimental studies in healthy volunteers and patient samples, whether there is scientific evidence supporting potential efficacy and safety of low doses of psychedelics in the treatment of affective disorders.

## Methods

**Figure 1.**
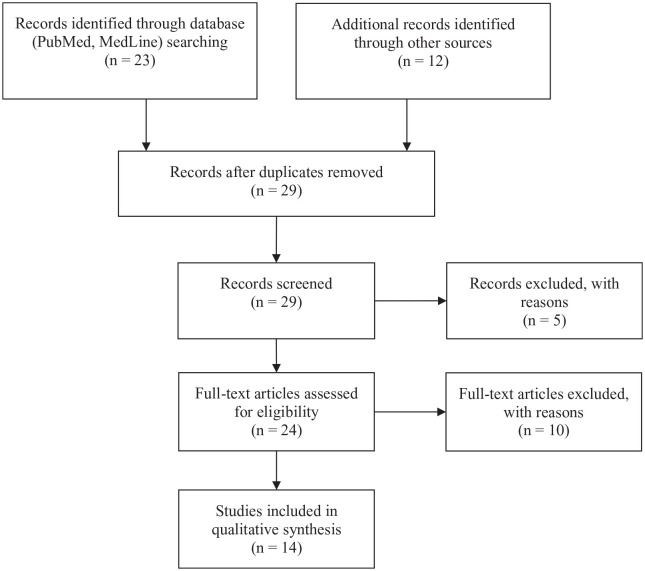
The selection and review process that resulted in 14 articles for inclusion in the current review.

In order to answer that question, a search string consisting of keywords from (1), (2), and (3), combined with the Boolean command ‘AND’ was used to search title and/or abstract. Search words were (1) depressive disorder or depression or unipolar depression or bipolar depression or dysthymic depression or chronic depression or neurotic depression or persistent depressive disorder or treatment-resistant depression or therapy-resistant depression or refractory depression or mood disorder or affective disorder; (2) microdosing or micro-dosing or low dose or mini dose, (3) psychedelics or classical psychedelics or hallucinogens or lysergic acid diethylamide or LSD or psilocybin or magic mushrooms or psilocybin truffles or psilocin. Searched databases PubMed and MedLine yielded 23 hits in total. De-duplication (*n* = 6) and removal of irrelevant articles (*n* = 5) reduced the number of articles to 12. The irrelevant articles either used a full psychoactive dose, or radiolabeled LSD, a low dose of dimenthyltryptamine (DMT), or ‘LSD’ referred to ‘Late Sleep Deprivation’ instead of the psychedelic substance, or preclinical research. With regard to preclinical research, this was not included because of the questionable generalizability to humans. A series of 12 relevant articles were localized in a book about microdosing psychedelics (*n* = 4),^27–34^ and a review paper describing the long-term effects of psychedelics (*n* = 1) and in my personal database (*n* = 7) was added.^35^ The oldest articles (*n* = 4) were harvested from the bibliographic database of the Multidisciplinary Association for Psychedelic Studies (https://maps.org/resources/psychedelic-bibliography), the rest (*n* = 8) *via* google scholar; 10 papers were excluded as they were based on claims of microdosers and not experimental research. These papers were not included in the review but were used in the introduction of this paper. Finally, this resulted in a final dataset of 14 experimental studies in humans (Figure 1). The findings of research with LSD and psilocybin are discussed in two separate sections; the methodological details of reviewed studies is presented in Tables 1 and 2.

**Table 1.** Methodological details of the included experimental studies with low doses of LSD.

Author	Aim, to test. . .	Design (number of conditions)	Intervention (Dosage, route of administration)	Sample (*n*)	Average age	Time of admin	Setting	Measures (findings)
Abramson *et al.*^36^	the mental effects of a range of LSD doses	Not stated; Supposedly a mixed within-between, some participants received multiple doses, though it was not clear who (6 dosing categories)	LSD (0, 1–25, **26–50, 51–75, 76–100, 101+** mcg, p.o.)	Non-psychotic, adult volunteers (31)	Not stated	Not stated	Laboratory and private office	Questions related to 5 mental state ‘themes’: euphoria (±, inverted U, peak at 51–75 mcg), dysphoria (–, peak at 51–75 mcg), perception and psychotic behaviour (–, dose-related increase), neurotic behaviour (–)
Bershad *et al.*^37^	the effect of low LSD doses on subjective experience and cognitive behaviour	Within-group design (4 drug conditions)	LSD tartrate (0; 6.5; 13; 26 mcg), single dose	Healthy volunteers (20)	25	9:30 AM	Private living room–style laboratory room (with a couch, table, and computer) Between tests participants could relax, read, or watch movies	Questionnaires: drug (+) and affective (+) effects, Computer tests: Working memory (–), Cognitive performance (–), Emotion recognition (–), Social inclusion (–), Convergent thinking (–), Physiological effects (+)
Bershad *et al.*^38^	the neural effects of a low dose of LSD on resting-state CBF and connectivity using functional magnetic resonance imaging	Within-group design (2 drug conditions	LSD tartrate (0, 13 mcg)	Healthy volunteers (20)	25	9:30 AM	Scanner	Questionnaires: Drug (+) and affective effects (+), Physiological effects (+), fMRI: connectivity analysis (+)
Family *et al.* ^27^	the safety, tolerability, pharmacokinetics, and pharmacodynamics of repeated low dose LSD administration	Mixed within-between group design (4 drug groups); Repeated dosing (dose each 3 days; 21 days in total)	LSD tartrate (0, 5, 10, 20 mcg)	Healthy volunteers (12/group)	62.9	Not stated	In-patient setting, no details were provided	Questionnaires: Drug (+), Physiological effects (–), Computer tests: Reaction time (–), Visual memory and learning (–), Visual attention (–), Spatial working memory (–), Balance and proprioception (–)
Gasser *et al.* ^39^	the safety and efficacy of LSD-assisted psychotherapy	Double-blind (2 drug conditions)	LSD free base (20, **200** mcg, p.o.)	Patients with anxiety associated with life-threatening diseases (11)	51.7	No stated	Safe, quiet, pleasant room in a private practice office; the participant could choose to lay down on a mattress on the floor or sit on a chair	Physiological measures (–) and adverse events (+, apparent, compared with the high dose was that more participants reported anger, anxiety, and abnormal thinking), self-report questionnaire for state and trait anxiety (+, state and trait anxiety increased the low dose group and decreased after the 200 mcg crossover)
Greiner *et al.* ^40^	the dose-response effects	Double-blind, not clear how many participants received more than one dose	LSD (0, 4, 7, 12, 20, **40** mcg, p.o.)	Healthy male volunteers (14); 0 mcg (3); 4 mcg (2); 7 mcg (6); 12 mcg (2); 20 mcg (6); 40 mcg (5)	Not stated	Not stated	Dark room, 20 min of each hour could be spent how they wanted	Mood (+; 7–40 mcg) and perception (body, alertness, emotion thought) (+, 20–40 mcg); Physiological measure [Galvanic skin conductance (+, 7 mcg), Pupil diameter (+, 12–40 mcg)]; Psychiatric information: changes in mood (cycles of depression, euphoria) and behaviour (+, 7–40 mcg)
Isbell *et al.* ^41^	I. the dose-response effects; II. the reproducibility of the LSD effect using a specific measure; III. The tolerance to LSD after (long-term) daily intake (4 sub-experiments; only 2 with low doses)	I. Within-Subject; II. Within-Subject; III. Within-Subject	I. LSD (0, 0.25, **0.5, 0.75, 1, 1.5, 2 mcg**/kg, p.o., intervals of 1 week); II. LSD (**60 mcg**, p.o.); IIIa. LSD twice a day for three days (10 mcg, 20 mcg, **30** mcg, or placebo, p.o.), day 4: LSD (**75** mcg, p.o.), 3 days placebo (or LSD, p.o.), day followed by LSD (**75** mcg, p.o.); IIIb. LSD, once daily for 7 days (20–**75** mcg, p.o.)	Former morphine addicts, all male, abstinent for at least 3 months; I. *n* = 8; II. *n* = 11; IIIa. *n* = 11; IIIb. *n* = 12	Not stated	I. No stated; II. Not stated; IIIa. First dose: 9 AM, second: 9 PM; 75 mcg: 9 AM (this was repeated in week two, groups shifted form LSD to placebo and VV); IIIb. 9 AM	Closed ward devoted to clinical research; they had their own room but were allowed to leave this in between the observations to mingle with other patients in a common dayroom	Mental effects questionnaire and observation (–, I.; +, IIIa–b. Reversible tolerance to LSD effects after dosing for three days in a row; tolerance disappeared after 3 days of abstinence), physiological effects (pupil size, blood pressure) (–, I.), Knee jerk (–, I.)
McGlothlin *et al.* ^42^	the mental effects of a high dose of LSD	Mixed Within-Between group, Within: 5 test days (baseline, 3 experimental sessions, 2 follow-ups at 2 weeks and 6 months; Between: Treatment (3 drug conditions)	LSD (25, **200** mcg), Amphetamine (20 mg; 5 immediate + 15 sustained release)	Healthy volunteers (*n* = 24/group)	Not stated	8:00 AM	Large, tastefully decorated room, specially designed to enhance the drug experience; music was played during most of the session	Anxiety: questionnaires and GSR, Attitude, Value, Aesthetic sensitivity, Creativity *(seen the nature of statistics that were performed, no findings are presented)*
Yanakieva *et al.*^33^	the effects of repeated low doses of LSD on time perception	Mixed within-between group design (4 groups); Repeated dosing (dose each 3 days; 21 days in total)	LSD tartrate (0, 5, 10, 20 mcg)	Healthy volunteers (12/group)	62.9	Not stated	In-patient setting, no details were provided	Questionnaires: Drug (+), Computer test: Time reproduction (+)

(+) = presence, (–) = absence of low dose LSD effect relative to placebo or another control condition; bold doses are not considered a microdose and are not considered in the last column where the effects are shown relative to the reference condition.

CBF, cerebral blood flow; LSD, lysergic acid diethylamide.

**Table 2.** Methodological details of the included experimental studies with low doses of psilocybin.

Author	Aim, to test. . .	Design (number of conditions)	Intervention (Dosage, route of administration)	Sample (*n*)	Average age	Time of admin	Setting	Measures (findings)
Griffiths *et al.*^28^	or to compare the ascending and descending sequences of drug dose exposure	Within-group design (5 drug conditions)	Psilocybin (0, 5, **10, 20, 30** mg/70 kg)	Healthy volunteers (18)	46	Not stated	aesthetic living-room-like environment with two monitors present; couch, eye mask, headphones for music	Questionnaire: Drug effects (+), Physiological effects (+)
Hasler *et al.* ^29^	or to explore the potential dose–response relationship of psilocybin on various (neuro-) psychological and physiological parameters	Within-group design (5 drug conditions)	Psilocybin (mcg/kg bodyweight, 0, 45, **115, 215, 315**)	Healthy volunteers (8)	29.5	Not stated	Psychiatrist present during the session	Questionnaire: Drug (+) and affective (–) effects, Paper-and-pencil test: Concentration (–); Physiological effects (–)
Madsen *et al.*^30^	the relationship between the subjective psychedelic experience, plasma psilocin levels and 5-HT2AR occupancy in the human brain	Within-group design (repeated measures of participant receiving a specific dose)	Psilocybin (3, **6, 12, 15, 18, 24, 30** mg)	Healthy volunteers (8)	33	Not stated	Two psychologists providing interpersonal support were present; During PET scans music was played	Positron Emission Tomography: 5-HT2A receptor binding (+), Questionnaire: Drug (+)
Moreno *et al.*^31^	the safety, tolerability, and clinical effects of psilocybin in patients with OCD	Within-group design (4 drug conditions)	Psilocybin (25, **100, 200, 300** mcg/kg, baseline)	OCD patients (9)	40.9	8:30 AM	Participants were asked to wear eyeshades, listen to music and minimize the interaction during the session; trained sitters were present	Questionnaire: OCD Symptoms (+)
Prochazkova *et al.*^32^	the cognitive-enhancing potential of microdosing psychedelics	Within-group design, Naturalistic study	Psilocybin truffles: average dose of 0.37 g (0.6 mg psilocybin baseline]^ⱡ^	Healthy volunteers (38)	31.1	Not stated	non-laboratory environment (microdosing event); tasks were conducted in a group setting free from outside distraction	Paper-and-pencil test: Intelligence (–), Convergent (+) and divergent (+) thinking

(+) = presence, (–) = absence of low dose psilocybin effect relative to placebo or another control condition; bold doses are not considered microdose and are not considered in the last column where the effects are shown relative to the reference condition.

ⱡ In This naturalistic study, dried psilocybin-containing truffles were taken by participants.

5-HT2A, 5-hydroxytryptamine 2A; 5-HT2AR, 5-HT2A receptor; OCD, obsessive-compulsive disorder; PET, positron emission tomography.

## Experimental research with LSD

In the past, a number of experimental LSD studies were conducted, investigating the effects on cognitive and physiological measures. Some of those studies included low doses of LSD and are described in detail here.^36,40–42^ Next to that, five recent studies were identified and included here; these aimed to assess the effects of a microdose LSD on cognition, subjective perception and brain activity.^27,33,37–39^ Given that the methodology of older studies did not always meet current standards, more methodological detail is provided for these studies, so that findings are interpreted in that specific context.

### Older research with LSD

Abramson and colleagues, who conducted a range of experiments with LSD in the 1950s, combined data from 141 experimental sessions with 31 participants with the aim of providing a clear view of the mental effects caused by different doses of LSD. As participants received different doses and observers used different questions, they made six ‘dose’ groups and clustered the symptoms into five classes: euphoria, dysphoria, changes in perception, neurotic behaviour and psychotic symptoms. Relevant for the current review is that eight participants were administered a dose between 1 and 25 mcg LSD. While findings showed a dose-related increase in psychotic behaviour and distortions in perception, this behaviour was not affected by the low dose of LSD. Dysphoria seemed to be relatively stable over doses, with a peak after a dose of 51–75 mcg LSD. Euphoria followed an inverted-U pattern with also a peak around 51–75 mcg LSD. Neurotic behaviour was found to be non-discriminant as it increased after administration of all doses, including placebo. The effects of low doses of LSD on the selected parameters therefore seem to be very mild or placebo-like.^36^ As the authors also stated, the findings should be regarded as descriptive due to the confounding factors of different measurements, settings, doses and the small sample size for some doses.

Isbell and colleagues published the findings of six experiments in which a range of LSD doses (0.25–2 mcg/kg or 10–180 mcg) was administered in several regimens, aiming to investigate the dose-response effect, the test-retest value of a series of mental and physiological measures, and tolerance after repeated doses of LSD.^41^ The latter was assessed in four studies, of which only two also included low doses (10–20 mcg) next to higher, psychedelic, doses of LSD. Of note, these findings were also published 1 year earlier, though less methodological detail was provided.^43^ The test-retest questionnaire was assessed after administration of 60 mcg LSD. Only the findings of the three studies administering low doses of LSD are described here. These all included self-rated and observed mental effects (e.g. perception/hallucinations, confusion/insight, nervousness, anxiety) and physiological measures (e.g. pupil size, blood pressure). The main findings were that (1) LSD produces dose-related effects with the exception of the lowest dose (0.25 mcg/kg), which did not produce differentiating effects from placebo; and (2) repeated administration of low doses (10–30 mcg), twice daily for 3 days produces a transient tolerance to the mental effects of a subsequent higher dose of LSD (75 mcg).^41^ Based on these findings, it can be suggested that daily microdosing is not efficient, but also that an abstinence period of 3 days is long enough to reinstate the mental response to a higher dose (75 mcg) of LSD.

Greiner *et al.* conducted a dose-effect study with five different doses of LSD and placebo administered to 14 healthy male volunteers. While they mentioned it was a double-blind design, they did not describe how many doses participants received, which was more than one as they stated that three participants received placebo, two 4 mcg, six 7 mcg, two 12 mcg, six 20 mcg and five 40 mcg LSD. Effects on self-rated mood and perception (of e.g. thoughts, body image), physiological measures, and observed mood and psychomotor behaviour were measured at least up to 4 h after treatment. The authors confirmed the ‘threshold dose status’ of 20 mcg of LSD, which it already had ‘by general consensus’.^40^ Participants noticed effects on mood starting from 7 mcg, but they did not experience the changes in mood states that were observed by the experimenter, including the cycling pattern of depressed and euphoric mood states.^40^ Linked to that, the authors expressed their concern about mood changes potentially negatively affecting higher-order cognitive processes like planning and motivation.^44^ Of note, no statistical analyses were performed and, looking at the sample size per dose and the way effects were reported, i.e. it was marked as a change when it was seen in more than 50% of the group, this paper should merely be seen as qualitative, descriptive research.

McGlothlin *et al.* aimed to test the long-lasting effects of repeated (3×) administration of a high dose of LSD (200 mcg) on measures of anxiety, attitude and value, aesthetic sensitivity, creativity and personality in healthy volunteers. Two control groups were administered single doses of amphetamine (20 mg) or LSD (25 mcg) on three separate occasions. Volunteers were tested at baseline, after administration of the treatment, and at 2 weeks and 6 months post-treatment. While the authors decided to combine the two control groups as the findings of both groups allegedly did not differ systematically, not much can be concluded about the difference in effects between the low (25 mcg) and high dose (200 mcg) of LSD.^42^ What can be inferred is that LSD (25 mcg) has a similar effect pattern as the stimulant amphetamine (20 mg) in the mentioned doses. Interestingly, throughout their paper, the authors give percentages of people in the three groups that have experienced specified effects acutely, at 2 weeks and 6 months follow up. The LSD (25 mcg) was labelled as ‘pleasant’ by the majority (78%) and without lasting effects (65%); LSD (200 mcg) was labelled as ‘dramatic and intense’ (71%) with some lasting effects (42%). Of note, statistics were performed on the aforementioned dimensions; however, the findings are not reported here for a number of reasons. First, as already stated, data of the two control groups were combined, which makes the findings less interesting for the current review as no direct statistical comparisons were conducted between the low and high LSD dose. Second, because of the selectivity of included participants in the analyses, for example for some analyses only participants who stated to have long-lasting effects at the 6 month follow up were included, for other comparisons, only a selection of the control group was taken ‘to compensate for the higher baseline scores in the LSD high dose group’. Of note, the 25 mcg LSD group was included as a control group in the hope they would experience enough visual or auditory hallucinations and therefore realize they had received LSD, which would be a good control for prior expectations. The same proportion of people in the LSD 25 mcg and amphetamine group thought they received LSD on one or more sessions.^42^

### Recent research with LSD

#### Studies in healthy volunteers

In a randomized, double-blind, placebo-controlled study, elderly subjects received different doses of LSD tartrate (0, 5, 10, and 20 mg) repeated (6 times) every 4 days for a period of 21 days in a between-group design (*n* = 12/group). Cognition was tested at different times and with different measures. The only statistically significant effect was an overestimation of time intervals of 2000 ms (and longer) in a time perception task after the fourth dose. These effects were most pronounced for the 10 mcg dose. The absence of effects on other cognitive processes is seen as evidence that there was no general disturbance of cognition but a specific effect on selective attention.^33^ Blood was collected to determine LSD concentrations after doses 1 and 6. It was shown that while LSD plasma concentrations were not detectable after 5 mcg, they were after 10 and 20 mcg; concentrations peaked approximately half an hour after administration. The total blood concentration after dose 1 and dose 6 did not differ, which demonstrates that this parameter is not affected by repeated doses. The average half-life over all data points was 8.25 ± 7.5 h, which is comparable with that of a full psychedelic dose (200 mcg), that is, 8.9 ± 5.9 h.^45^ Compared with the placebo group there were not significantly more adverse events in the LSD groups; however, one more often reported effect in the LSD group was a mild-to-moderate headache. The authors argued that the intensity was not of such an order that it would disrupt daily tasks.^27^

Bershad *et al.* investigated the effects of three doses of LSD tartrate (6.5, 13 and 26 mcg LSD, corresponding to 5, 10 and 20 mcg of base LSD) in a placebo-controlled within-subject study on subjective experience and cognitive measures.^37^ Subjects felt under the influence after taking 13 and 26 mcg LSD. They also felt better, friendlier and more anxious compared with placebo. In addition to an increase in ‘liking’ the substance, ‘disliking’ also increased. No effects were found on other mood states (vigor, depression, anger, confusion or fatigue), cognitive skills or social behaviour. The latter two were assessed with tasks sensitive to the effects of full psychedelic doses of a psychedelic.^46,47^ While no persisting effects on mood were shown 2 days after administration, it has to be noted that this questionnaire was completed by only 55% of the participants.^37^ A low LSD dose led to a statistically significant, though clinically irrelevant, increase in systolic (13, 26 mcg) and diastolic (26 mcg) blood pressure 2 h after LSD administration, compared with placebo. There were no differences in heart rate and basal body temperature after intake of LSD compared with placebo.^37^ This study shows that repeated administration of low doses of LSD can be regarded as safe on the parameters assessed, though the authors suggest focussing on heart parameters in future studies due to concerns about potential 5-HT2B receptor-mediated ECG abnormalities after repeated use.^48^

In a recent functional imaging study, participants underwent a scan session 90 min after taking placebo and LSD tartrate (13 mcg). Findings revealed an LSD-induced change in brain connectivity in the limbic (‘emotion’) system. More specifically, the connectivity between the amygdala and the angular and frontal gyrus increased, while connectivity with the superior temporal gyrus decreased. The increase in connectivity was related to the changes in positive mood, measured by the Positive and Negative Affect Schedule (PANAS).^49^

#### Study in patients with anxiety

Gasser and colleagues investigated the safety and efficacy of LSD-psychotherapy in patients with anxiety related to life-threatening diseases. Next to the experimental group who received LSD 200 mcg twice, they included an active control group receiving 20 mcg of LSD. This low dose was thought to produce short-lived, mild LSD effects that would not substantially facilitate the therapeutic process. Two regular psychotherapy sessions followed each LSD session. The control group entered an open-label crossover to 200 mcg LSD after the treatment blind was broken. The number, frequency and intensity of drug-related adverse events was higher in the high dose condition compared with the low dose condition, though anger, anxiety, and abnormal thinking were more frequent in the low dose condition. LSD did not affect physiological parameters. Self-rated anxiety (trait and state) decreased after two high dose sessions with LSD – an effect that was sustained up to 12 months after treatment. In the low dose group however, anxiety increased after two sessions with LSD 20 mcg, and decreased after the open-label crossover to LSD 200 mcg – an effect that was also measurable at 12 months follow up. Of note, the total sample size was very small, and the low dose was only give to three participants. The authors noted correctly that the (fluctuating) medical conditions of the participants could have influenced the psychological state; hence, the self-rated anxiety.^39^ While the data seem to suggest a low dose of LSD does not support the therapeutic process, this study did not aim to test this hypothesis, and future studies in larger samples should corroborate this.

## Experimental research with psilocybin

In total, five studies were identified testing the effects of (low) doses of psilocybin on subjective experience and cognitive performance in healthy volunteers and patients with OCD.^28–32^ The methodological details of those studies are presented in Table 2 and described in the following.

### An uncontrolled, naturalistic study

One of the included experimental studies was an uncontrolled, naturalistic study, in which a group of people who self-administered psilocybin-containing truffles were tested in an informal social setting. This study showed that convergent (*n* = 27; 0.41 g truffles) and divergent (*n* = 33; 0.35 g truffles) thinking improved 1 h and a half after taking the truffles compared with a pre-measurement.^32^ Given the uncontrolled nature of this study, placebo-controlled experimental studies are needed to be able to say with certainty whether these effects are due to the intervention and not to learning effects, expectation, or the social context.^32^

### Controlled, experimental studies

Hasler and colleagues tested the effects of different doses of psilocybin on subjective experience and cognition. The lowest dose (45 mcg of psilocybin per kg of bodyweight), which would qualify as microdose (2.3 mg of psilocybin for a 70 kg-person), caused a decrease in heart rate 6 h after ingestion. Other than that, no significant differences between this dose and placebo were detected.^29^ Griffiths and colleagues demonstrated mild psychedelic effects after administration of a 5 mg/70 kg bodyweight dose of psilocybin compared with placebo.^28^

In a recent positron emission tomography study, Madsen and colleagues demonstrated that psilocybin (3–30 mg) binds to the 5-hydroxytryptamine 2A (5-HT2A) receptor, and that the degree of receptor occupancy (%) is related positively to the intensity of the psychedelic experience.^30^ The lowest dose (3 mg) led to a psychedelic experience of average (40%) intensity; the 5-HT 2A receptor occupancy rate was 43%; the highest psilocybin dose (30 mg) led to a psychedelic experience of maximum intensity (100%) and a receptor occupancy of 65%.^30^

### Study in obsessive compulsive disorder patients

Moreno and colleagues reported findings of their small-scaled study in which they administered on separate occasions a range of psilocybin doses to patients (*n* = 9) with obsessive compulsive disorder. Besides a low dose of psilocybin (25 mcg/kg bodyweight = 1.75 mg/70 kg), three higher doses were included (100, 200, 300 mcg/70kg). Symptom reduction after treatment with a low dose of psilocybin relative to baseline was demonstrated.^31^ This suggests that a very low dose of psilocybin could cause a better balance between habitual behaviour and cognitive control, something that might also be relevant in depressed patients.^50^ Nonetheless, future studies in (large) patient samples have to confirm this.

## Discussion

The purpose of this article was to investigate scientific evidence for the therapeutic potential and safety of microdosing psychedelics for depression. To that end, (placebo-controlled) experimental studies testing the effects of low doses of LSD or psilocybin on psychological and cognitive processes in humans were reviewed. Both LSD and psilocybin were shown to have no, to very subtle, effects on mood state, selective cognitive processes (time perception, convergent and divergent thinking), and brain regions involved in affective processes.^32,33,40^ While low LSD doses were experienced as pleasant,^37,41^ it was also shown that drug disliking and anxiety increased,^37,39^ and that a cycling pattern of depressive and euphoric mood changes can occur.^40^

It is as yet unclear whether psychedelic microdosing is of therapeutic value for depression due to the limited amount of studies (with small sample sizes) that have been conducted. Nonetheless, the aforementioned effects on selective cognitive processes, resembling in a milder way the effects of full psychedelic doses,^51,52^ and without impairing cognitive processes,^47^ suggest that low doses of psychedelics could play a role in depression. Some underlying cognitive mechanisms of action, deduced from the observed effects, that is, increased divergent thinking and slowed down time perception, could be the induction of respectively increased cognitive flexibility,^31,32^ and the production of a heightened experience of ‘being more in the present’ or mindful,^33,53^ which could lead to lessened ruminative thinking and more self-compassion, decreasing depressive symptoms.^54,55^ With regard to its safety, it was demonstrated that low doses are well tolerated and have no-to-minimal effects on physiological parameters.

### The cause of effects: expectancy and underlying biology

Despite the positive media coverage on microdosing, and its effects on social behaviour, creativity, and productivity,^56^ reviewed scientific evidence demonstrates that the effects are not that pronounced, as one would expect. This might indicate that expectancy determines large part of the effect in users. However, a study in users set out to test the role of expectancy in the effects of microdosing surprisingly showed that the reported effects were not the expected effects.^20^ In addition, people really expecting certain effects sometimes stop with microdosing because the practice was not deemed effective, or the effects did not meet their expectations.^14^

With regard to placebo-controlled studies, the presence of a placebo can correct for this expectancy effect. However, a placebo effect might occur, as shown by Olson *et al.*, which can make it harder to find subtle effects as these expectancy or ‘placebo’ effects might decrease the chance of demonstrating statistically significant differences between the active treatment and placebo.^57^ While in all the reviewed studies the chance that participants would receive LSD or psilocybin was equal to, or above 50%, the effect pattern, with selective effects on specific measures stems positive, in that the demonstrated effects are ‘real’ and due to the administered substance rather than created by expectation.

Future studies could consider not revealing beforehand the exact substance participants will receive, though rather present a list with options of substances they could receive,^58^ or use additional ‘active’ treatments to control for expectancy and placebo effects.^42^ While there are benefits to this approach, limitations are the increased study costs, the expected higher attrition rate when using a within-subject study with more conditions, and the increased complexity of statistical analyses. In addition, future studies might also want to compare the effects of a range of low doses between groups of people who have experience with the use of psychedelics, and those who are drug-naive, as previous experience might increase the sensitivity to detect changes in for example, mood state. An older study already showed that participants who were trained to recognise the effects of psychedelics were able to do this.^58^ This potentially lowered detection threshold in experienced users would imply they need less of the substance or conversely, drug naïve (patients) would need a higher microdose to experience equal effects.

While this (microdosing) psychedelics research field is still in its infancy, preliminary findings indicate that low doses of both LSD and psilocybin affect assessed biological processes.^30,38^ A first study showed changed connectivity in brain areas involved in affective behaviour, after a single dose of LSD (13 mcg tartrate, p.o.). Interestingly these changes were also related to positive mood effects,^38^ which suggests this might be a potential mechanism underlying alleged therapeutic effects in depression. Of note, studies need to confirm (the persistence of) these effects in patient populations since this study was conducted in healthy volunteers, and the effect at brain and behavioural level was assessed at the acute state, not when the drug left the bloodstream.

Madsen and colleagues who investigated the 5-HT2A receptor occupancy rate after a single-dose administration of psilocybin, showed that the lowest psilocybin dose (3 mg, p.o.), which would be regarded as a microdose, was related with a receptor occupancy rate of 2%.^30^ As this change from baseline is obviously very small, and labeled as ‘non-substantial’ by the authors, future studies could elucidate whether repeated administrations in a larger sample, in contrast to this single administration in one person, produce a different and/or larger effect at receptor level, and potentially at a behavioural level too. Interestingly, in this light was the tolerance to perceptual effects of a higher dose of LSD after three repeated low doses LSD administered on consecutive days,^43^ which suggests adaptation at receptor level. In addition, while psychedelics are usually labeled as (partial) 5-HT2A agonists, they also activate, though to a lesser extent, other 5-HT receptors (e.g. 1A), and other neurotransmitter systems such as the dopaminergic and adrenergic.^4,23^ Mechanistic studies blocking these pathways, and including assessments of cognitive performance and emotional state, will reveal the respective contributions of these neurotransmitters and respective receptors to the psychedelic-induced effects and potential therapeutic mechanism.

### Adverse effects

While there is no mention in the media about possible negative effects related to microdosing psychedelics,^20^ users do report to experience negative effects when asked. These are in general limited to the dosing days when physical discomfort and increased feelings of anxiety can arise.^15,18,59^ Some others mentioned that they had unpleasant ‘free’ days,^59^ something that was also raised by an author suffering from depression, seeking help in microdosing psychedelics as self-treatment. She stated that microdosing felt as a relief, a treatment for her depression, although sometimes she also had days when she was more irritated than on other days.^60^ In the reviewed experimental studies, a single acute dose was generally well tolerated by healthy volunteers. Repeated doses did not produce more adverse effects than placebo, although mild headache was mentioned more often after microdoses of LSD, compared with placebo.^27^

In terms of physiological effects, no LSD-related effects on heart rate of basal body temperature were assessed after a single dose of LSD.^37^ Family and colleagues demonstrated a clinically irrelevant increase in systolic (13 and 26 mcg) and diastolic (26 mcg) blood pressure, measured 2 h after LSD compared with placebo.^27^ Family and colleagues do advise, despite the apparent safety of low doses of LSD, to include monitor heart parameters after repeated doses of LSD in follow-up studies.^27^ This was mentioned due to concerns about substances that act on the 5-HT2B receptor, and that in the past caused abnormalities of the heart valves after repeated intake, though of doses that exceeded the microdose range.^61^ In addition, when conducting clinical trials in depressed patients, tapering off serotonergic antidepressants under medical supervision will be necessary, as no information about potential interactions is available. Nonetheless, this would require careful weighing up of the risks of discounting serotonergic antidepressants against the potential benefits of using serotonergic psychedelics in microdoses.

### Lessons learned and future perspective

#### Psychological support

A first point of attention is the fact that both in anecdotal reports of microdosers, and in findings of reviewed experimental studies, increased anxiety (during intoxication) is mentioned.^15,18,39,40^ This suggests that, although a microdose does not produce a psychedelic experience, psychological support is needed to regulate increased anxiety. Looking into the details of the settings of previous studies, it is also shown that most of the times the setting was a safe, warm environment, in which support was standing by. Future clinical trials in depressed patients should therefore consider, for example, to not send patients home after they have been administered their microdose as anxiety might arise. An interesting note was that the presence of anxiety might signify latent emotions coming to the surface, something that could accelerate a healing process in a therapeutic context, as these emotions can then be discussed with the therapist if deemed necessary by the patient.^22^ This support might not only have to be limited to the dosing day as previously mentioned, users also can experience less pleasant dose-less days.^59^ This psychological support might then also stimulate or contribute to an enhanced state of mindfulness and (hence) cognitive flexibility, thereby facilitating the therapeutic process.^54,62^

#### Dose and dosing schedule

A second point of discussion is the ‘effective’ dose, and the associated dosing schedule. The reviewed studies do not provide robust evidence in favour of a specific dose, but rather give a range in which psychedelics show subtle (beneficial) effects without producing extreme perceptual distortions; for LSD (base) this is between 10 and 20 mcg of LSD, and for psilocybin between <1 and 3 mg.^16^ An important addition when talking about LSD doses is that, especially in this microdosing range, it is essential to specify whether LSD base or LSD (‘salt’) tartrate is administered. Liechti clarified this in a commentary: ‘a dose of 100 µg LSD base corresponds to 123 µg LSD tartrate’.^16^ Apparently, the older research used LSD tartrate, whereas modern research, with full psychedelic doses, uses LSD base.^4^ The recent microdosing studies in this review all used LSD tartrate,^27,33,37,38^ except for one.^39^

With regard to the dosing schedule, only one recent study aimed to test the effects of repeated LSD doses on psychological and cognitive functions.^27,33^ It was shown that LSD blood concentrations were not affected after repeated dosing when leaving two dose-free days in between. Of note, previously it was demonstrated by Isbell and colleagues that tolerance to the effects of (a higher dose of) LSD (75 mcg) occurred after repeated dosing with low doses of LSD (10–30 mcg), given twice daily for 3 days in a row.^41^ Nonetheless, this tolerance was transient and disappeared after three dose-less days. Based on this information, it is tentatively concluded that daily dosing, something that is not common practice amongst users, will probably be not efficacious, while 2–3 days in between would already be sufficient to curb the tolerance. This reminds of the ‘Fadiman’ schedule, in which users are recommended to use according to a three day cycle, with 1 day ‘on’, and 2 days ‘off’ the substance to experience the effects of the microdose on day one and two, and to use the third day to experience, and remind yourself of how the ‘normal’ situation is, without microdosing.^13^ Usually users repeat this cycle a couple of times, with a pattern of microdoses for years being rather uncommon.^13,15,18^ However, only research can show the persistence of the effects and the necessity to microdose psychedelic for a prolonged time. Preliminary findings with full psychedelic doses demonstrated remission from depression after one or two doses,^63^ with the quality of the psychedelic experience being predictive in the therapeutic outcome.^12^ Future research can test the efficacy of low *versus* higher doses of psychedelics in the treatment of depression, and the longevity of therapeutic effects and its predictors.

#### LSD or psilocybin: does it matter?

A third point to be addressed by future research is to compare the effect pattern of LSD and psilocybin in repeated low doses, in one study. While users sometimes attribute more cognitive enhancing and/or stimulating effects to LSD, psilocybin is associated with more ‘soft’ emotional or well-being effects.^17,22^ One older study,^58^ not included in this review, shows that participants sometimes confuse low doses of psilocybin with LSD. Interesting here is that these participants were trained to recognize and discriminate the effects of these substances. The reason why this paper was not included is because the concrete effects experienced by the participants were not included and therefore could not be used to answer the question of the present review.^58^ This research suggests that the effects of psilocybin and LSD in low doses can be similar. In addition, findings from another study suggest that LSD (25 mcg) indeed induces stimulant effects, as the effects were similar to those of amphetamine (20 mg).^42^ This does not exclude the possibility that psilocybin and LSD would have dissimilar effects; it rather supports the claims by users that LSD in low doses has stimulant effects.^17,22^ Therefore, in light of therapy with low doses of LSD or psilocybin it is necessary to know whether they have a different, and perhaps a complementary, effect pattern that could be employed successively to treat different symptoms (‘cognitive’ or ‘affective’) observed in one psychiatric disorder.

## Conclusion

While preliminary findings demonstrated the therapeutic efficacy of full psychedelic doses in the treatment of depression, anecdotal reports suggest that lower doses, without the psychedelic experience, are beneficial too. As clinical microdosing trials in depressed patients yet have to take place, some of the reviewed studies showed subtle positive effects on cognitive and affective processes that are dysfunctional in depressed patients. Of note, because this is based on small samples of mostly healthy, young volunteers, it is too early to draw conclusions about its therapeutic efficacy. Nevertheless, these preliminary findings warrant the exploration of the safety and therapeutic efficacy of microdosing psychedelics for depression.
